# Mimicking Strategy for Protein–Protein Interaction Inhibitor Discovery by Virtual Screening

**DOI:** 10.3390/molecules24244428

**Published:** 2019-12-04

**Authors:** Ke-Jia Wu, Pui-Man Lei, Hao Liu, Chun Wu, Chung-Hang Leung, Dik-Lung Ma

**Affiliations:** 1 State Key Laboratory of Quality Research in Chinese Medicine, Institute of Chinese Medical Sciences, University of Macau, Macao 999078, China; Yb67513@um.edu.mo (K.-J.W.); Mb85817@um.edu.mo (P.-M.L.); 2 Department of Chemistry, Hong Kong Baptist University, Kowloon Tong, Hong Kong 999077, China; yjhxliuhao@163.com (H.L.); ccwuchem@gmail.com (C.W.)

**Keywords:** protein–protein interactions, virtual screening, mimetics, drug discovery

## Abstract

As protein–protein interactions (PPIs) are highly involved in most cellular processes, the discovery of PPI inhibitors that mimic the structure of the natural protein partners is a promising strategy toward the discovery of PPI inhibitors. In this review, we discuss recent advances in the application of virtual screening for identifying mimics of protein partners. The classification and function of the mimicking protein partner inhibitor discovery by virtual screening are described. We anticipate that this review would be of interest to medicinal chemists and chemical biologists working in the field of protein–protein interaction inhibitors or probes.

## 1. Introduction

Protein–protein interactions (PPIs) are involved in the regulation of biological processes, including cell proliferation, signal transduction, transcription, and apoptosis [1]. Since numerous ailments are associated with abnormal PPIs, the inhibition of PPIs is an attractive approach for the generation of new therapeutics. However, because of their large and amorphous interfaces, targeting PPIs is a great challenge in pharmaceutical and academic research.

In recent years, computer-aided approaches became useful tools to assist scientists in drug discovery. In particular, virtual screening emerged as a complementary technique to aid high-throughput screening (HTS) in pharmaceutical development. Virtual screening can reduce the number of compounds to be screened in bioassays, leading to a large reduction of time and cost [2,3]. Virtual screening strategies can be traditionally classified into two broad types: ligand-based virtual screening (LBVS) and structure-based virtual screening (SBVS). LBVS strategies include approaches such as pharmacophore-based methods, quantitative structure–activity relationships (QSAR), and three-dimensional shape matching [4]. On the other hand, SBVS techniques mainly revolve around the docking of molecules to three-dimensional (3D) structures of the biological target as determined by X-ray crystallography, nuclear magnetic resonance (NMR), or homology modeling.

Recently, virtual screening found increasing use for identifying inhibitors against various targets. Sun et al. constructed QSAR models of Sirtuin 1 (SIRT1) ligands and discovered 12 compounds as inhibitors of SIRT1 through ligand-based virtual screening [5]. Yang et al. identified a potent and selective KDM5A inhibitor using structure-based virtual screening [6]. Wu et al. reported mitoxantrone as an inhibitor of NAE using virtual screening of an approved drug database [7]. Virtual screening was also used to develop inhibitors of PPIs. Yang et al. described a compound as an inhibitor of the VHL–HIF1α interaction using structure-based virtual screening [8]. Zhong et al. utilized structure-based virtual screening and identified a cytosine alkaloid compound as an inhibitor that inhibited the menin–MLL interaction and another compound as a potential inhibitor of TLR1–TLR2 heterodimerization [9,10]. However, virtual screening still has many challenges and limitations that need to be overcome, especially its high false positive rate, which limits virtual screening to initial screening only [11].

**Figure 1:**
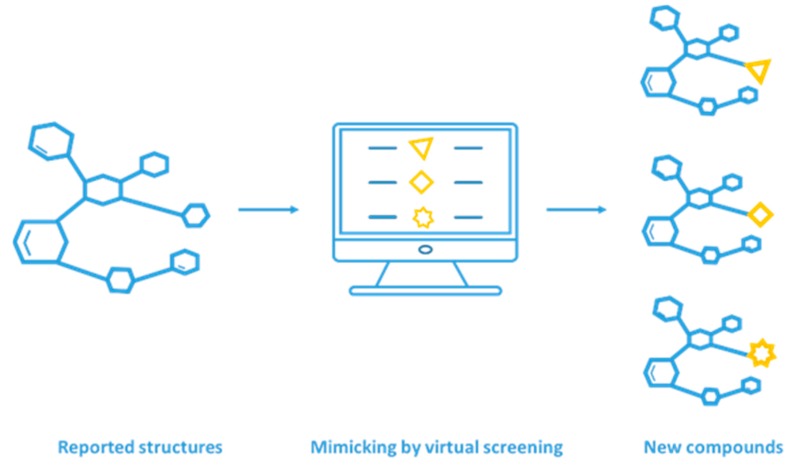
Mimicking strategy for inhibitor discovery by virtual screening [31]. (Reprinted with permission from Copyright (2015) Wiley—VCH Verlag GmbH & Co. KGaA.).

The rational design of compounds that mimic key interactions at the protein–protein interface is another successful strategy for PPI inhibitor discovery [12]. Compared with low-molecular-weight compounds, mimetics can be more selective and show lower toxicity. Modification and optimization of peptide mimetics can also improve structural stability, resulting in increased oral availability [13]. For example, Groß et al. designed and synthesized a soluble peptide mimicking CXCR4 to interrupt the gp-120 and CXCR4 interaction [14]. In this context, the combined utilization of mimicking strategies and virtual screening has broad application prospects for drug discovery (Figure 1). Based on this strategy, many mimetics targeting cancer-related PPIs were discovered using virtual screening, such as p53 mimetics for the MDM2–p53 interaction [15,16,17,18,19,20], BH3 mimetics for the Bcl-2–BH3 interaction [21,22,23,24,25,26], and SMAC mimetics for the IAP–SMAC interaction [1,27,28,29]. Knowledge of the 3D structures of proteins allows the use of different approaches for mimetics design. For the interruption of PPIs, inhibitors may be larger in size than traditional drug compounds. Peptides and proteins mimetics are increasingly considered to be viable therapeutics for PPI inhibitor discovery [30]. However, there are still some challenges in protein or peptide mimetics design. For example, we still do not fully understand folding and the physical forces that stabilize protein structures. Moreover, sequences with many degrees of freedom can complicate the sequence search, which leads to a requirement for effective methods to find sequences related to a particular structure and measure essential protein folding criteria.

In this review, we discuss the recent advances in the application of virtual screening to design protein or peptide mimetics for PPI inhibitor discovery, and we summarize different methods for virtual screening. The classification of mimicking strategies and the function of the mimicking protein partner inhibitors discovered using virtual screening techniques are also described.

## 2. Integrating Mimicking and Virtual Screening Strategy for Protein–Protein Interaction Inhibitor Discovery

Recent studies indicated that certain types of PPIs are amenable for targeting by small molecules, which can block the interaction between a protein and its peptide or protein partner via binding at the proteinous interface [7,32,33,34,35,36,37,38]. As key residues of the protein or peptide may serve as a beginning point for PPI inhibitor design, the effective mimicking of peptides in their biologically active conformation and the development of mimicking ligands are important goals in science beyond the development of PPI modulators. Meanwhile, virtual screening emerged as a complementary technique to aid mimicking strategy in pharmaceutical development, complementing high-throughput screening (HTS) techniques. Integrating mimicking and virtual screening is a potentially viable strategy for protein–protein interaction inhibitor discovery. In this section, we describe the application of virtual screening and a mimetic strategy for PPI inhibitor discovery and discuss their merits and drawbacks.

### 2.1. Virtual Screening for PPI Inhibitor Discovery

Virtual screening is a kind of computer-aided technique that is usually considered as an initial step in the lead discovery process in order to enrich the library with active compounds and predict experimental activity [11]. Usually, based on the information about the target or the ligands of reported compounds, virtual screening is usually classified into two types: ligand-based virtual screening (LBVS) and structure-based virtual screening (SBVS). LBVS strategies depend on the similarity or dissimilarity of the compounds of interest, and they require a large amount of structure–activity data from a large chemical compound library. One of the ligand-based approaches is QSAR modeling, which focuses on achieving a correlation between the physicochemical and structural properties of the ligands and their biological function and potency. QSAR modeling includes two-dimensional (2D-QSAR) and three-dimensional QSAR (3D-QSAR). Scientists use 2D-QSAR and 3D-QSAR properties of ligands to build up a model of biological activity, which can be applied to predict the activity of some new compounds [39]. Compared with 3D-based algorithms, 2D-based algorithms are usually faster but may be less accurate; moreover, 2D-based algorithms cannot find new active compounds with dissimilar chemical structures [40]. Moreover, 2D- and 3D-QSAR do not consider ligand conformations, protein structure and flexibility, or solvation effects. Another LBVS approach is based on the similarity of compounds, which is a simple computational method with low cost, focusing on obtaining compounds that are similar to known ligands. However, this method is easily influenced by human users because it is difficult to objectively select the input molecules [41]. It is usually carried out by using common chemical features from the 3D structures of some known ligands that represent interactions between the ligands and the target. Pharmacophore modeling is another approach for LBVS. Based on analyzing the structures of known inhibitors against a target, a ligand-based pharmacophore can be generated that describes the spatial arrangement of chemical features of active compounds. However, under many circumstances, it is hard to find a library with functionally and structurally diverse molecules with quantitative activity data for a given protein. More importantly, the lack of publications with negative results hinders the identification of inactive molecules, resulting often in the development of qualitative common feature pharmacophores only from active compounds [41,42]. Finally, as LBVS applications are generally based on the properties of the known ligands, the diversity of the hits discovered are generally limited.

In contrast, SBVS techniques do not require knowledge of the biological activity of known compounds. Instead, 3D structures of the protein must be known or inferred. Protein-ligand docking is widely applied to identify compounds that are predicted to bind tightly to the active sites of the target. During the SBVS process, the 3D structure of a target protein and a set of ligands are considered as starting points and screened by virtual filtering, followed by docking and scoring to identify potential lead candidates. Many algorithms were developed to perform SBVS, such as DOCK, GOLD, And AUTODOCK, which can be used for identifying the binding mode and binding affinity between protein and ligand [43]. After docking and scoring, a set of compounds with the highest predicted binding affinity against the target can be obtained [44]. One relatively new strategy that falls within the purview of SBVS is the binding site comparison approach. This strategy, which can be utilized for drug repurposing and polypharmacology, relies on the recognized fact that many different proteins have similar binding sites [45]. Thus, binding sites in any given protein can be searched and matched with specific chemical structures. Finally, the pharmacophoric approach can also be employed within SBVS. If a high-resolution 3D structure of the target is available, a structure-based pharmacophore of the binding site can be generated based on the structural features of the binding site. In this case, a library of known inhibitors against the target is not needed. Compared to LBVS (Table 1), SBVS is more likely to identify new scaffolds because it is based on physical interactions calculated in silico, rather than relying on the similarity/dissimilarity of known ligand compounds. Thus, SBVS may be able to identify inhibitors with unique mechanisms of action [11,46]. Another difference is that, unlike LBVS, the docking model obtained by SBVS can be used for interaction analysis, in order to further enhance the affinity or selectivity of the compounds.

Receptor and ligand flexibility is crucial for predicting drug binding and evaluating thermodynamic and kinetic properties. Molecular dynamics (MD) simulation is a technique for investigating atomic and molecular motion, and it is widely and effectively used for analyzing the relationship between the structure and function of molecules [47,48]. The main advantage of MD simulations is that they can thoroughly sample the conformational space around both the protein and the ligand under realistic conditions, accommodating both structural flexibility and entropic effects, thus allowing the thermodynamics and kinetics of the drug–target interaction to be more accurately calculated [47]. Therefore, MD simulation can be combined with SBVS or LBVS to further understand the binding mode of candidate molecules, thus accelerating the process of drug development [49]. Additionally, MD simulations can be used to identify potential pockets and binding hotspots of PPIs [50]. Saez et al. used MD simulations to predict the atomic interactions of the PcTx1–cASIC1 interaction and the hotspot residues of their interface, which could be beneficial for designing therapeutically useful PcTx1 mimetics [51]. By combining structural information, MD, and functional experiments, they obtained detailed insight into the molecular basis of this PPI. The TRAF6–Basigin interaction is implicated in melanoma metastasis. Biswas et al. used MD simulations to study the interactions between individual proteins and TRAF6–Basigin complexes, revealing conformation changes in the PPI and the adoption of a helical conformation [52].

In terms of the chemical library used for SBVS or LBVS, different filters can be used. For fragment-sized compounds, Congreve et al. described a “rule of three” with molecular weight < 300, logP < 3, number of hydrogen bond donors and acceptors < 3, and number of rotatable bonds < 3 [53]. Alternatively, based on physicochemical properties, the “Pfizer’s Rule of 3/75" can be applied to predict the toxicology of compounds. Compounds with calculated partition coefficient (ClogP) < 3 and topological polar surface area (TPSA) > 75 are approximately 2.5 times more likely to be safe in in vivo assays [54]. However, overly strict application of filters may introduce bias, leading to the exclusion of potentially active compounds. To assess the potency of the hits derived from virtual screening, IC_50_, EC_50_, K_i_, or K_d_ values can be calculated. Ripphausen considered four subdivisions of potency (<1, >1–10, >10–100, and >100 μM), and suggested that docking hits are generally weakly potent, falling into the 1–100 μM range [55].

As virtual screening is often used as first stage of the primary screening process, false positives are a common problem. Based on six cases, Schierz et al. reported that the average percentage of false positives from the high-throughput primary screen is quite high at 64% [56]. To eliminate false positives, cross-referencing between primary and confirmatory screening assays is required. On the other hand, selectivity is a crucial aspect for developing potent PPI inhibitors. Off-target effects can arise when the compounds bind to other protein targets rather than their intended target, leading to side effects [57]. Virtual screening can be used for resigning, repositioning, and predicting side effects or toxicity of drugs, which can significantly decrease the time and cost of development compared to the traditional drug discovery process [58,59]. In one example, Spahn et al. used a computational simulation to create chemical modifications of fentanyl, an opioid pain killer with severe adverse effects due to off-target effects throughout the body. The newly discovered compound, named NFEPP, possesses a lower pKa and eliminates pain by selectively activating the MOR pathway in the inflamed acidic area without causing side effects [60].

Taken together, virtual screening greatly decreases the time and money costs by processing thousands of compounds in a short time in silico, thereby reducing the number of compounds to be synthesized or purchased [61]. However, because virtual screening relies on analyzing the physicochemical properties of compounds rather than biological activity directly, it has a high rate of false positives or false negatives compared to cellular or phenotypic screens [62].

In order to improve the efficiency of screening for more bioactive PPI lead inhibitors, the mimicking peptide strategy can be used. Virtual screening can be employed to target PPI surfaces or to mimic “hotspot” residues. Peptide mimetics that mimic the bioactivity of the parent peptides can also show improved pharmacokinetic and pharmacodynamic properties, such as bioavailability and stability [63]. It should be noted that the mimicking protein domain can be achieved with protein backbone scaffolds or small molecules [30,31].

### 2.2. Structure-Based Mimicking Peptide Strategy for PPI Inhibitor Discovery

Peptides are utilized as feasible molecules to mimic protein binding sites [64]. Peptidomimetics are non-peptide compounds that mimic the conformation and characteristics of peptide molecules to interrupt PPIs [63]. In general, chemical synthetic, screening, and structure design approaches are usually used for designing and extending the diversity of peptide-derived chemical structures, as well as enhancing their metabolic stability [64]. Synthetic strategies can explore and expand the chemical space for peptidomimetics. Screening strategies, including high-throughput screening and fragment screening, are often used to identify hot hits and discover peptidomimetics based on reported compounds [65]. Meanwhile, design strategies can use hotspot residues as starting points to design analogues by mimicking key secondary-structure motifs involved in the PPI interface. Design strategies can be subdivided into sub-structure search, *de novo* design, and bioisostere design [65]. As peptides usually contain secondary structures such as α-helices and β-sheets, peptidomimetics for inhibiting PPIs should be able to mimic these structures in order to be able to displace the natural peptides [66]. Therefore, peptides that mimic α-helices or β-sheets of proteins are attractive targets for drug discovery. α-helix structures are indispensable secondary structural elements which constitute most structured protein domains and contribute greatly to the protein–protein interface. Main strategies for synthesizing α-helix mimetics include (i) cross-linking of peptide side chains and the incorporation of stabilizing caps at the N-terminus, (ii) use of foldamers to modulate backbone variations, and (iii) introduction of projecting rod-like elements that mimic the side chains of an α-helix [31,67]. For example, Ernst et al. designed a polyamide foldamer as an α-helix mimetic, leading to the synthesis of inhibitors of the Bak BH3/Bcl-xL complex [68]. Meanwhile, a β-strand is an extended structural element between three and 10 amino acids long, and adjacent β-strand structures can be connected laterally backbone hydrogen bonds, forming a twisted or pleated sheet. β-sheets play key roles in maintaining the tertiary and quaternary structures of proteins, as well as PPIs. A number of strategies were utilized to design β-strand and β-sheet mimetics: (i) incorporation of turn mimetics to nucleate β-sheet generation, (ii) macrocyclization via covalent or noncovalent linkages, and (iii) introduction of β-strand-enforcing residues [31]. In addition to α-helix structures and β-strand structure mimetics, mimicking the turn structure of peptides is another potential approach for PPI inhibitor discovery. Turn structures are anomalous secondary structures that differ from α-helix and β-sheet structures due to the non-repetitive dihedral angles of the main chains. Turn motifs allow a peptide chain to fold back, and are important for forming globular proteins [31,69,70]. For instance, Bartfai et al. designed a β-turn mimetic that interrupted the interaction between IL-1RI and MyD88 in the TIR domains [71].

Gimeno and co-workers developed a classification of peptide mimetics depending on the extent of similarity to the native peptide. Class A mimetics contain the parent peptide amino-acid sequence, with the side chains being arranged to closely mimic the active conformation of the native peptide. Class B mimetics possess further modification of the native sequence, including the introduction of non-natural amino-acid residues, other small molecular motifs, or changes of the backbone sequence. Class B mimetics include foldamers, β- and α/β-peptides, and peptoids. Class C mimetics are highly modified structures with small molecular motifs and changes in the main chains of the peptide. Class D mimetics mimic the method of action of the native peptide rather than through structural mimicry of the side chains, and they can be developed via affinity optimization of class C mimetics or, alternatively, they can be identified by virtual screening [31]. An alternative classification of peptide mimetics was also described in the past two decades. Type I mimetics are short peptide sequences that mimic the α-helical motif of a PPI interface. Type II mimetics are functional mimetics that are based on a small molecular scaffold rather than a peptide scaffold. Type III mimetics include non-peptide templates that mimic the topography of the original helix by retaining the spatial arrangement of key binding residues [31,72,73]. In peptide mimetics design, one of the main challenges is that the topological shapes of proteins are complex, leading to variations in the types of interactions, binding pockets, and recognition sites formed [74]. This variability of PPIs is a crucial aspect that has to be mastered for the design of peptide mimetics targeting PPIs.

### 2.3. Integration of Mimicking Strategies with VS for PPI Inhibitor Discovery

**Figure 2:**
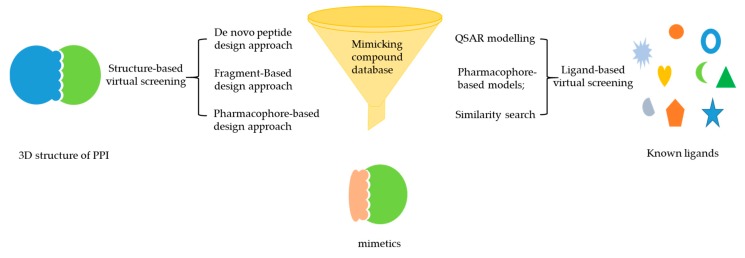
Different approaches for mimetics discovery based on structure-based virtual screening (SBVS) and ligand-based virtual screening (LBVS) [78]. (Reprinted with permission from Copyright (2002) Elsevier Science B.V.).

Effectively mimicking the bioactive conformation of a peptide is a critical part of developing mimics as PPI inhibitors. However, developing mimetics with appropriate pharmacokinetic properties is a key challenge to overcome [31]. To strike a balance for these two properties, applying virtual screening can allow for simultaneous optimization of affinity and pharmacokinetic properties [75,76,77]. Thus, the integration of mimicking strategies and virtual screening is a complementary strategy for efficiently developing PPI inhibitors. In this section, we introduce and classify mimicking strategies using different virtual screening approaches (Figure 2).

#### 2.3.1. *De Novo* Peptide Design Approach

*De novo* peptide design is an attractive approach for constructing designed peptides with desired structures and functions, including peptide mimetics targeting PPIs. *De novo* design can create novel molecules that do not exist in known compound databases. This method only requires a scaffold library and a few key anchor residues as a starting point. By knowledge of the structural features of the native peptide, new inhibitors with the desired secondary structural characteristics can be built up *de novo* according to the targeted binding site. The virtual *de novo* peptide design method can be considered to comprise the following stages: (i) documentation of key anchor residues and the preparation of the scaffold library, (ii) virtual screening to find scaffold fragments that the anchor residues can be attached to, (iii) sequence design and structure refinement, and (iv) experimental validation [79]. There are several advantages of *de novo* peptide design to developing protein mimics. The first one is that the backbone of the natural protein sequence can be utilized as a template to initiate the design. Another advantage is that knowledge of sequence/structure relationships and/or statistical forcefields from native proteins can be used to guide the sequences of the designed peptides [80]. Therefore, *de novo* peptide design approach is a complementary strategy for peptide mimetics discovery.

Using the joint application of the *de novo* peptide design approach and peptide mimetics design, Li et al. designed PD-1-binding peptides by mimicking five residues (Y56, R113, A121, D122, and Y123) of the ligand PD-L. The most potent peptide Ar5Y_4 had a K_D_ value of 1.38 ± 0.39 μM, which was comparable to the binding affinity of the PD-L1. Ar5Y_4 showed the ability to interrupt the binding of PD-L1 to PD-1, providing a potential strategy for further optimization of PD-L1 peptide mimetics [74,81]. Smadbeck et al. used a three-stage *de novo* peptide design approach to design EZH2 inhibitory peptides. The approach comprises a sequence selection stage, a fold specificity calculation stage, and an approximate binding affinity calculation stage. The novel peptide SQ037 showed the highest in vitro response, with an IC_50_ of 13.5 μM. Compared to the native and K27A mutant control peptides, SQ037 had greater potency as an inhibitor and showed higher specificity to EZH2 [79]. Ruiz-Gómez et al. optimized the *de novo* design approach for small scaffolds mimicking protein recognition epitopes of large, non-structured, and discontinuous PPIs. They applied this novel re-scaffolding approach to the *de novo* design of potent interleukin 10 (IL-10) ligands that mimic the high-affinity receptor IL-10R1 [82]. Overall, these studies demonstrate that computer-aided *de novo* design is an effective strategy for peptide mimetics discovery.

#### 2.3.2. Fragment-Based Design Approach

The fragment-based design approach uses fragments with low molecular weight and small size as starting points for modifying into high-affinity compounds. Compared with HTS, the fragment-based design approach can result in higher hit rates and a higher probability of synthesizing an efficient binding compound [83]. Hence, fragment-based approaches are particularly effective for generating small molecules or peptides targeting PPIs. The most common biophysical screening techniques for the fragment-based design method include differential scanning calorimetry (DSF), ligand- or protein-based nuclear magnetic resonance (NMR), surface plasmon resonance (SPR), isothermal titration calorimetry (ITC), or X-ray crystallography [1,74]. The fragment-based design approach is widely applied to mimetics design. For example, Petros et al. discovered a high-affinity ligand for the anti-apoptotic protein Bcl-X_L_ using fragment-based screening. From NMR-based structural studies and parallel synthesis, a potent BH3 mimetic ligand was obtained, which bound to Bcl-xL with an inhibition constant (K_i_) of 36 ± 2 nM [22,23]. Using NMR-based screening, parallel synthesis, and structure-based design, further modification of this ligand was achieved by Oltersdorf and co-workers. They discovered ABT-737, a small-molecule inhibitor of the anti-apoptotic proteins Bcl-2, Bcl-X_L_, and Bcl-w, which showed two to three orders of magnitude more potent affinity than previously reported compounds [24]. Although ABT-737 showed great antitumor activity in murine tumor xenograft models, it lacked oral bioavailability. Park et al. reported that targeted modifications at three positions of ABT-737 led to a 20-fold improvement in the pharmacokinetic/pharmacodynamic relationship (PK/PD). The resulting compound ABT-263 was orally available in a xenograft model of human small-cell lung cancer, and induced complete tumor regressions in all animals [25]. Based on ABT-263, Souers et al. redesigned and reported the first-in-class orally bioavailable Bcl-2-selective inhibitor with potent anticancer activity in vitro and in vivo [26]. Fragment-based design, in concert with computational approaches, show high promise for peptide mimetic design and discovery.

#### 2.3.3. Pharmacophore-Based Design Approach

Pharmacophore-based approaches enables the virtual screening of large numbers of peptide mimetics using a conventional pharmacophore broadly derived either using structure-based or ligand-based methods, depending on whether a 3D structure of the target is available or not [74,84]. The pharmacophore-based approach can be divided into four steps: (i) atom typing, (ii) conformational sampling, (iii) hypothetical pharmacophore construction, and (iv) virtual screening of candidate ligands against the hypothetical pharmacophore [85]. Using the pharmacophore approach, Hansen et al. synthesized small beta-peptidomimetics with anti-staphylococcal activity. Their research showed that small β-peptidomimetics can mimic the antimicrobial activity of much larger antimicrobial peptides (AMPs), making them promising candidates for treating bacterial infections [74,86]. Caporuscio et al. developed compounds that showed micromolar potency against replication of HIV-1 in cells via a target-based pharmacophore model mapping the CD4-binding site on HIV-1 gp-120 [87]. Hall et al. conducted a two-round computational screening of potential peptide mimetic compounds in order to develop inhibitors of the ανβ3 integrin receptor. Biological testing revealed that the peptide mimetic molecules potently inhibited hantavirus with two thousand times more potency than the natural cyclic peptide (cyclo-[CPFVKTQLC]). The second round of screening furnished molecules with improved chemical diversity by building up the pharmacophore models [74,88]. Atatreh et al. started with a 3D pharmacophore and performed virtual screening to discover a series of MDM2–p53 interaction inhibitors with inhibition activity at the submicromolar level, which showed anticancer activities against different breast cancer cell lines [89]. Overall, the pharmacophore-based design approach is a suitable method for peptide mimetics discovery when the target protein structure is unavailable.

#### 2.3.4. Integration of Mimicking Strategies with LBVS for PPI Inhibitor Discovery

In contrast to structure-based approaches, LBVS uses the structures of known binders as templates to discover and identify diverse bioactive compounds with high affinity. In general, LBVS methods depend on the application of computational descriptors of molecular structure, properties, or pharmacophore features, and they analyze relationships between active and database or test compounds in various defined chemical descriptor spaces [90,91]. Three major methods are usually utilized for LBVS: QSAR modeling, pharmacophore modeling, and the efficient similarity method. QSAR modeling can be broadly divided into three steps: (i) collect compound data, (ii) develop and validate QSAR models, and (iii) use the models to identify compounds from a chemical library. QSAR approaches are not computationally intensive, so they can be performed relatively quickly [92]. However, ligand-based 2D- and 3D-QSAR models do not consider ligand conformations, protein structure and flexibility, or solvation effects, which may lead to poor accuracy. When the three-dimensional (3D) structure of a target is unknown, pharmacophore modeling can be utilized to identify biologically active compounds via chemical features such as hydrogen bonding and lipophilicity as the input data for flexible alignment. Ligand-based virtual screening involves two different methods: (i) flexible alignment of molecules by considering only the atomic contributions, and (ii) use of other chemical features that are unrelated to 3D pharmacophore representations, such as hydrogen bonding and lipophilicity [84,93]. In the similarity method, compounds with similar structures are assumed to have similar activities, allowing the topological, steric, electronic, and/or physical properties of compounds to be predicted by comparison with known molecules [94]. Six types of similarity are exploited: chemical similarity, molecular/2D similarity, 3D similarity, biological similarity, global similarity, and local similarity [95]. In recent years, LBVS was increasingly applied to identify active compounds as PPI inhibitors [96]. Švajger et al. used two parallel virtual screening methods targeting the TLR4–MD-2 interface by mimicking interactions with MD-2 to discover novel TLR4 antagonists. They identified a potent hit compound with an IC_50_ value of 16.6 μM and no cytotoxic properties, which may be a potential agent to treat sepsis and neuropathic pain [97]. Varney et al. previously reported the interaction of Lipid II with defensins, based on the 3D structure of the human defensin peptide HNP1–Lipid II complex. They designed a pharmacophore model and used it for screening for defensin mimetics, leading to the first Lipid II-targeted low-molecular-weight compound, BAS00127538 [98]. Ambaye et al. used the co-crystal structure of a lead peptide antagonist and combined a shape-based similarity search, molecular docking, and 2D-similarity searches to identify nine novel phenylbenzamide-based antagonists of the Grb7 SH2 domain as potential Grb7 anticancer therapeutics [99].

## 3. Conclusions

Due to the critical roles of PPIs in disease, targeting PPIs is a potential therapeutic strategy. High-throughput screening is a widely used technique in drug discovery; however, a large investment into compounds and screening assays is required. Virtual screening is an emerging technology for drug discovery because there is no need for physical compounds and bioassays for screening. As we highlighted in this review, virtual screening is used for discovering mimetics of PPIs based on different approaches, such as *de novo* peptide design approach, fragment-based design approach, pharmacophore-based design approach, and ligand-based design approach. We anticipate that the integration of virtual screening with mimicking strategies will become a powerful tool in cancer research and that that this review could arouse the interest of chemical and biological scientists working in the field of PPI inhibitors.

## Figures and Tables

**Table 1:** The advantages and limitations of structure-based virtual screening (SBVS) and ligand-based virtual screening (LBVS). QSAR—quantitative structure–activity relationships.

	Types	Pros	Cons
SBVS	1) Pharmacophore-based models	Uses protein structure	Increased screening time
	2) Molecular docking	Not biased toward existing ligand structures	Higher false positives
	3) Binding site comparisons	Takes protein flexibility into consideration	Oversimplification of scoring functions
LBVS	1) Similarity methods	Simple and fast	Requires existing ligands
	2) QSAR modeling	Less computationally intensive	Poor accuracy
	3) Pharmacophore-based models	Protein structure information may remain unknown	Lack of consideration of protein structural framework
